# Changes in Hyaluronan Metabolism and RHAMM Receptor Expression Accompany Formation of Complicated Carotid Lesions and May be Pro-Angiogenic Mediators of Intimal Neovessel Growth

**Published:** 2008-05-12

**Authors:** Jerzy Krupinski, Priya Ethirajan, M. Angels Font, Marta Miguel Turu, John Gaffney, Pat Kumar, Mark Slevin

**Affiliations:** 1 Department of Neurology, Stroke Unit, Hospital Universitari de Bellvitge (HUB), and IDIBELL, Barcelona, Spain; 2 Centro de Investigación Cardiovascular, CSIC/ICCC, Hospital de la Santa Creu i Sant Pau, Barcelona, Spain; 3 School of Biology, Chemistry and Health Science, Manchester Metropolitan University, Manchester, U.K

**Keywords:** Hyaluronan, atherosclerosis, RHAMM, angiogenesis

## Abstract

Previous studies have shown that changes in expression of the glycosaminoglycan, hyaluronan (HA) were associated with erosion in areas of post-mortem coronary artery liable to rupture. Angiogenesis is an important feature of ulcerating haemorrhagic plaques prone to rupture. HA is a glycosaminoglycan known to possess potent angiogenic properties on metabolism to oligosaccharides of HA (o-HA) in the presence of hyaluronidase (HYAL) enzymes. In this study, we have examined HA receptor and HYAL enzyme expression in a series of carotid artery specimens used as vascular transplants and exhibiting various stages of atherosclerotic lesions as determined by anatomo-pathology. Our results demonstrated dramatically increased expression of HYAL-1 in regions of inflammation associated with complicated plaques. Receptor for HA-mediated motility (RHAMM), which is known to be important in transducing angiogenic signals in vascular endothelium, was strongly expressed on intimal blood vessels from complicated lesions but almost absent from other regions including adventitial vessels. Metabolism of HA, together with up-regulation of RHAMM in complicated plaque lesions might be partly responsible for over-production of leaky neovessels and predisposition to plaque rupture.

## Introduction

Myocardial infarction and stroke represent the second and third leading causes of death and disability in the Western world respectively. The majority of cases result from thrombosis or embolism following rupture of unstable coronary and carotid arterial plaques. Development of this pathology is thought to be as a consequence of a combination of a number of risk factors, including age, gender, hypertension, hyperlipidaemia, smoking, diabetes and infections. The atherosclerotic plaque is a dynamic structure that undergoes continuous remodeling of the extracellular matrix during development, and on which its structural integrity is dependent (Miguel et al. 2006). Lesions including phase 2 and upwards following endothelial injury are prone to rupture (Stary et al. 1995). Acute changes within the plaque including active matrix remodeling, intraplaque hemorrhage associated with vascularization and fibrous cap rupture are a prelude to the onset of clinical ischemic events, however, the mechanisms and activation processes leading to this are not fully understood. Angiogenesis occurs within vascular lesions, resulting in formation of a network of capillaries, which extend into the thickened intimal layer associated with atherosclerosis and inflammation, and ultimately increasing susceptibility of the plaque to rupture (Mofidi et al. 2002; Moulton, 2006; Krupinski et al. 2006). The identification of plaque erosion sites rich in proteoglycan expression and associated with vessel occlusion without rupture of the fibrous plaque cap represents a novel pathophysiological basis for atherothrombosis (Kolodgie et al. 2002; Hennerici, 2004).

Hyaluronan (HA) is a non-sulphated linear glycosaminoglycan consisting of repeating units of (α,1–4)-D-glucuronic acid-(β,1–3)-N-acetyl-D-glucosamine. HA is found in its native state as a high molecular weight polymer (>10^6^ kDa) in the extracellular matrix of almost all animal tissues and in significant quantities in the skin (dermis and epidermis) and the brain (Slevin et al. 2006). Apart from its role as an inert viscoelastic lubricant which is essential for healthy joint function (West and Kumar, 1991), HA has a crucial role in regulation of the angiogenic process. In particular, HA is a potent regulator of vascular endothelial cell (EC) function. Native high molecular weight HA (>10^6^ KDa) is synthesized by a family of enzymes called HA synthases (HAS), and is anti-angiogenic, inhibiting EC proliferation and migration (West and Kumar, 1989; Deed et al. 1997) as well as capillary formation in a 3D collagen gel model (Sattar et al. 1994). Degradation products of specific size (3–10 disaccharide units; o-HA) stimulate EC proliferation, migration, sprout formation and result in angiogenesis in the chick chorioallantoic membrane (Slevin et al. 2002, 1998; West et al. 1985). Generation of this ‘angiogenic’ o-HA, from the naturally occurring HA polymer is mediated by action of the endoglycosidase hyaluronidase (HYAL; Lokeshwar et al. 2001), in association with tissue damage, and inflammatory disease (West and Kumar, 1989). The biological functions of HA/o-HA are thought to be initiated through cell surface receptors (particularly, CD44 and RHAMM (receptor for HA mediated motility)), resulting in signal transduction activation and ultimately cell mitogenesis. In vascular EC, both CD44 (Nandi et al. 2000; Slevin et al. 1998) and RHAMM (Lokeshwar et al. 2000), have been identified as potential targets for transduction of o-HA-induced mitogenesis. We have previously demonstrated that o-HA but not native HA induced up-regulation of the immediate early response genes c-jun, jun B, Krox 20, Krox 24 and c-fos in bovine aortic EC (BAEC) (Deed et al. 1997). Similarly, o-HA induced rapid CD44 dependent activation of PKC, Raf-1 kinase, MEK-1 and ERK1/2 resulting in mitogenesis in these cells (Slevin et al. 2002).

Previous studies have demonstrated an increase in expression of hyaluronan in distinct regions of human post-mortem aortic atherosclerotic plaques (Kolodgie et al. 2002; Papakonstantinou et al. 1998), although the size of the expressed molecule was not determined. In this manuscript, we have investigated the expression of HA metabolising enzymes and receptors for HA in a series of carotid artery transplants.

## Methodology

### Carotid artery transplants

Carotid specimens were obtained from dying patients, used for vascular transplants and exhibiting various stages of the atherosclerotic disease process (Phase I–V). Plaque pathology and characteristics including ulcerating, haemorrhagic or eroding regions was determined by anatomo-pathology (Table 1).

### Western blotting

Antibodies to the HA receptors, TSG-6 and RHAMM as well as hyaluronidase 1 and 2 and HA synthase 1 and 2, were supplied by our collaborators (Dr. Katalin Mikecz, Chicago, U.S.A., Dr. Eva Turley, Ontario, Canada, Professor Rashmin Savani, Philadelphia, U.S.A. and Dr. Evi Heldin, Uppsala, Sweden respectively). Their activities and specificities have been confirmed previously in detail (AlQteishat et al. 2006 and references therein). Anti-CD44 was bought from Calbiochem (U.K.). Briefly, tissue samples (50 mg) were lysed and protein separation carried out using SDS-PAGE electrophoresis as described previously (Slevin et al. 1998). Blots were stained overnight at 4 °C with primary antibodies described above and (Sigma, 1:1000) used as a loading control. Protein concentration was estimated from the band intensity by densitometry. Results are not quantitative and are used only to demonstrate differences in expression as a basis for selection of IHC analysis. All experiments were performed twice and a representative example is shown.

### Immunohistochemistry

Cellular localization was examined by immunohistochemistry, The Avidin-Biotin-Peroxidase method (ABC Vectastain kit, Vector Laboratories, Peterborough, U.K.) was used for the qualitative demonstration of antigens in tissues. Antibodies described above were used at 1:50 dilution. De-parrafinised 5 μm sections were treated for 10min in a boiling solution of concentrated citric acid (pH 6.0; Vector Laboratories) in a pressure cooker to unmask the antigens. Sections were stained with primary antibodies for 2h at RT, with the appropriate HRP-conjugated secondary antibody for 1h at RT, and then counterstained with haematoxylin. Negative control slides had the primary antibody replaced with PBS or the appropriate IgG pre-immune serum. Specificity of the antibodies has been previously established (AlQteishat et al. 2006a and b).

## Results

### Complicated plaque regions expressed increased concentration of HYAL-1

**Figure 1:**
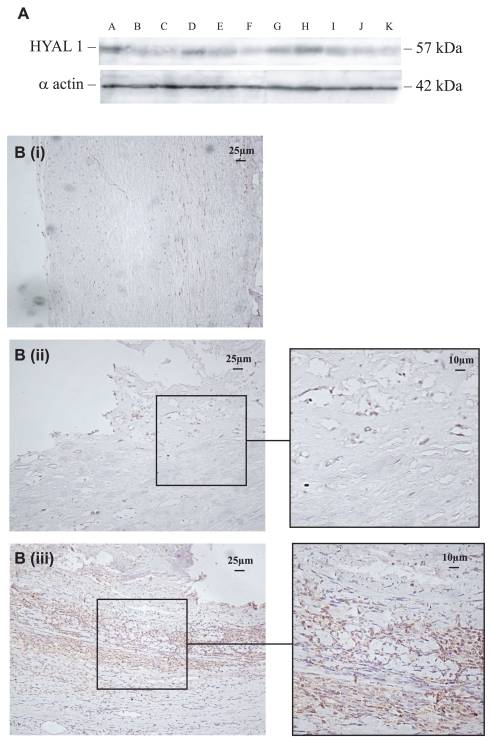
HYAL-1 expression was increased in complicated plaque regions. (**A**) Western blots showing relative expression of HYAL-1 in a series of carotid arteries obtained at endarterectomy (Table 1). Highest expression was seen in complicated plaques (A and H; Table 1). (**B**) IHC showed only weak expression of HYAL-1 in the media of normal looking vessels (i; B), but strong expression in the intimal neovessels (ii; A) and inflammatory regions (iii; H) of complicated plaques.

Weak expression of HYAL-1 was seen in Western blots of carotid transplants with no evidence of intra-luminal plaques and in those with stable fibrous structures (Fig. 1a; e.g. B and F and Fig. 1b (i); B shown). In contrast, complicated plaque lesions showed higher expression of HYAL-1 (Fig. 1a; e.g. A and H), particularly in areas of inflammation (Fig. 1b (ii) and (iii); A and H shown respectively). HYAL-2 was weakly expressed in normal looking arteries and no difference was seen in plaque lesions (data not included). Only weak expression of HAS1/2 was seen in both normal arteries and non-complicated/complicated regions with no significant differences in neovessel rich/poor areas of intima (data not shown).

### RHAMM receptor was identified in neovessels from complicated plaque lesions

**Figure 2:**
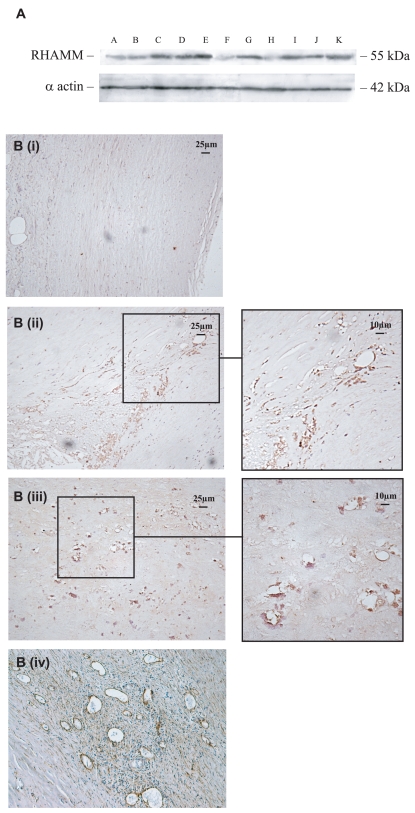
RHAMM expression was increased in complicated plaque regions. (**A**) Western blots showing relative expression of RHAMM in carotid arteries obtained at endarterectomy (Table 1). Highest expression was seen in complicated plaques (C and E; Table 1), but RHAMM was also expressed in non-complicated plaques (D and G). (**B**) IHC showed only weak expression of RHAMM in the media of normal looking vessels (I, B), but strong expression in the intimal neovessels (ii and iii; E) of complicated plaques. Endothelial cells of neovessels in complicated plaques demonstrated staining of CD105 (iv; E).

RHAMM receptor was weakly expressed in normal looking arteries as shown by Western blots (Fig. 2a; e.g. B and F). IHC confirmed a general lack of expression even in adventitial blood vessels (Fig. 2b (i); B shown). Carotid arteries with complicated lesions demonstrated increased staining in Western blots, and strong localization around plaque intimal neovessels (Fig. 2b (ii) and (iii); E shown). The presence of intimal blood vessels was confirmed by staining serial sections with antibodies to CD105 which stained active endothelial cells (Fig. 2b (iv); E shown). No differences in CD44 or TSG-6 expression were found (data not included).

## Discussion

New blood vessels may have an active role in plaque metabolic activity and actively promote its growth beyond the critical limits of diffusion from the artery lumen. Later in the progression of the disease, the inherent weakness of newly forming blood vessels could result in development of intra-plaque haemorrhage and instability (Mofidi et al. 2001; Herrmann et al. 2006; Krupinski et al. 2006). In this study, we have demonstrated that HYAL-1, which can depolymerise n-HA to angiogenic fragments, was increased in inflammatory complicated plaque regions. Previous studies have shown that HA was up-regulated in atherosclerotic lesions of apoE deficient mice, although the molecular weight was not determined (Cuff et al. 2001). In stable lesions, the fibrous cap is enriched with ECM molecules including versican and biglycan, which promote stability, viscoelasticity and inhibit vascular growth.

Rupture sites, however, have almost complete absence of ECM, in particular n-HA, possibly due to increased expression of matrix metalloproteinases (Kolodgie et al. 2002). It has been suggested that loss of n-HA corresponds with decreased integrity of the fibrous cap. Plaque erosion sites are distinct from rupture sites in that they contain many more SMC and ECM. These sites also tend to progress to thrombi. A significant increase in HA was found at the erosion site and at the plaque-thrombus interface of eroded vasculature, although the molecular size was not determined. Administration of either high molecular weight or angiogenic fragments (4–16 dissacharides) of HA reduced SMC proliferation and neointima formation in rats after balloon catheter injury suggesting a beneficial effect on developing lesions (Chajara et al. 2003). In this study, we found no evidence of increased HA synthesis in complicated angiogenic lesions.

In this paper, we show for the first time that the RHAMM receptor is over-expressed in neovessels from complicated plaque regions, and therefore might be responsible for enhanced endothelial cell activation via intracellular signal transduction following binding of o-HA. Evidence has shown that changes in expression of HA and its receptors occur concomitantly with transformation of stable to unstable arterial plaques. Increased expression of HA together with its receptor CD44 were found in rupture-prone areas in post-mortem coronary arteries (Farb et al. 2004), whilst increased expression of CD44 was found in atheromatous plaque microvessels, and treatment with anti-CD44/CD44v6 antibodies, reduced EC proliferation *in vitro* (Krettek et al. 2004). In contrast, we did not find any difference in expression of CD44 between non-complicated and complicated carotid plaques.

In summary, although n-HA is a strong inhibitor of blood vessel growth, and for example, treatment of experimental balloon catheter vascular injury was associated with an inhibition of neointimal formation (Savani and Turley, 1995), it is possible that at injury sites, enzymatic or oxidative/nitrative breakdown of n-HA into smaller fragments, stimulates the growth of new vessels and could encourage plaque haemorrhage and rupture. Thus, changes in metabolism of HA, together with cellular receptor expression might be responsible for key dynamic changes in plaque vulnerability.

Rupture or thrombosis of unstable atherosclerotic plaques in the coronary arteries is a major cause of heart infarction. Development of this disease occurs over a period of decades, during which time, the arterial tissue undergoes a remodeling process making it susceptible to rupture. Hyaluronan is an important component of the arterial matrix, and may be intimately associated with this process.

## Figures and Tables

**Table 1:** Anatomo-pathological characteristics of plaques used in study.

	Sex	Age	Plaque description
A	m	69	Advanced, Complicated with thrombosis, inflammation +++, angiogenesis, active, focal necrosis, calcification
B	m	68	Small plaque, lipidic core, inflammation ++
C	m	79	Advanced Complicated with thrombosis, rupture, calcification, inflammation +++, angiogenesis, active
D	m	75	Normal, type I, thickenning of intima
E	f	86	Advanced, active, lipidic core, inflammation +++, angiogenesis, focal necrosis
F	f	76	Normal, thickenning of intima
G	m	56	Normal, thickenning of intima
H	m	74	Advanced, complicated, calcified plaque, inflammation +, angiogenesis
I	m	70	Advanced, active plaque, lipidic core, inflammation +++, angiogenesis, focal necrosis, small calcifications
J	m	82	Small plaque, calcification, inflammation ++, angiogenesis, erosion
K	m	66	Advanced, complicated plaque, calcification, inflammation +++, angiogenesis, rupture, lipidic core
